# *N*′-[(*E*)-2-Hy­droxy-5-meth­oxy­benzyl­idene]pyridine-4-carbohydrazide monohydrate

**DOI:** 10.1107/S160053681301235X

**Published:** 2013-05-15

**Authors:** M. K. Prasanna, M. Sithambaresan, K. Pradeepkumar, M. R. Prathapachandra Kurup

**Affiliations:** a Department of Chemistry and Research Centre, PRNSS College, Mattanur 670 702, Kannur, Kerala, India; b Department of Chemistry, Faculty of Science, Eastern University, Sri Lanka, Chenkalady, Sri Lanka; c Department of Applied Chemistry, Cochin University of Science and Technology, Kochi 682 022, India

## Abstract

The title compound, C_14_H_13_N_3_O_3_·H_2_O, adopts an *E* conformation with respect to the azomethine bond and crystallizes in the amide form. An intra­molecular O—H⋯N hydrogen bond occurs. In the crystal, the lattice water molecule plays a major role in the supramolecular architecture by interconnecting adjacent molecules into a three-dimensional netwrok by means of O—H⋯O, O—H⋯N and N—H⋯O hydrogen-bonding inter­actions. The structure also features two non-classical C—H⋯O inter­actions.

## Related literature

For properties of carbohydrazide and its derivatives, see: Mangalam & Kurup (2011 ▶). For mol­ecular sensing of metals, see: Bakir & Brown (2002 ▶). For related structures and background references, see: Kargar *et al.* (2010 ▶); Shafiq *et al.* (2009 ▶); Sithambaresan & Kurup (2011 ▶). For the synthesis, see: Mangalam *et al.* (2009 ▶).
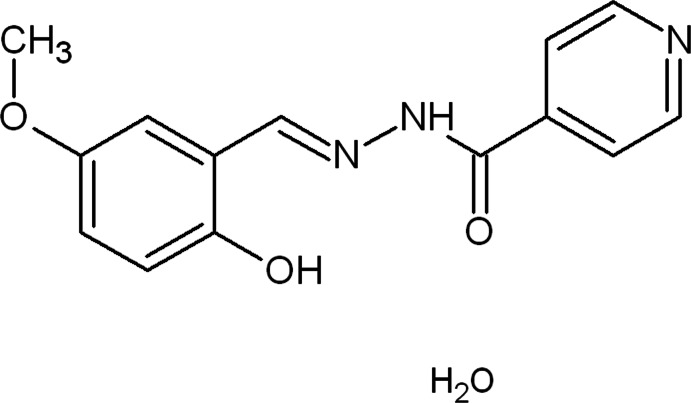


## Experimental

### 

#### Crystal data

C_14_H_13_N_3_O_3_·H_2_O
*M* *_r_* = 289.29
Orthorhombic, 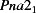

*a* = 12.6455 (16) Å
*b* = 12.7423 (16) Å
*c* = 8.9306 (9) Å
*V* = 1439.0 (3) Å^3^
*Z* = 4
Mo *K*α radiation
μ = 0.10 mm^−1^
*T* = 296 K
0.40 × 0.30 × 0.25 mm

#### Data collection

Bruker Kappa APEXII CCD diffractometer
Absorption correction: multi-scan (*SADABS*; Bruker, 2004 ▶) *T*_min_ = 0.965, *T*_max_ = 0.976
10294 measured reflections
1668 independent reflections
1134 reflections with *I* > 2σ(*I*)
*R*_int_ = 0.056

#### Refinement

*R*[*F* ^2^ > 2σ(*F* ^2^)] = 0.045
*wR*(*F* ^2^) = 0.124
*S* = 1.00
1668 reflections
208 parameters
6 restraints
H atoms treated by a mixture of independent and constrained refinement
Δρ_max_ = 0.17 e Å^−3^
Δρ_min_ = −0.15 e Å^−3^

### 

Data collection: *APEX2* (Bruker, 2004 ▶); cell refinement: *APEX2* and *SAINT* (Bruker, 2004 ▶); data reduction: *SAINT* and *XPREP* (Bruker, 2004 ▶); program(s) used to solve structure: *SHELXS97* (Sheldrick, 2008 ▶); program(s) used to refine structure: *SHELXL97* (Sheldrick, 2008 ▶); molecular graphics: *ORTEP-3 for Windows* (Farrugia, 2012 ▶) and *DIAMOND* (Brandenburg, 2010 ▶); software used to prepare material for publication: *SHELXL97* and *publCIF* (Westrip, 2010 ▶).

## Supplementary Material

Crystal structure: contains datablock(s) I, global. DOI: 10.1107/S160053681301235X/fj2628sup1.cif

Structure factors: contains datablock(s) I. DOI: 10.1107/S160053681301235X/fj2628Isup2.hkl

Supplementary material file. DOI: 10.1107/S160053681301235X/fj2628Isup3.cml

Additional supplementary materials:  crystallographic information; 3D view; checkCIF report

## Figures and Tables

**Table 1:** Hydrogen-bond geometry (Å, °)

*D*—H⋯*A*	*D*—H	H⋯*A*	*D*⋯*A*	*D*—H⋯*A*
N2—H2′⋯O1*S* ^i^	0.89 (1)	1.91 (1)	2.784 (4)	168 (4)
O1*S*—H1*A*⋯O3^ii^	0.86 (1)	1.92 (2)	2.764 (4)	170 (4)
O1*S*—H1*B*⋯N3^iii^	0.86 (1)	2.05 (2)	2.874 (5)	160 (4)
O2—H2*A*⋯N1	0.85 (1)	1.89 (3)	2.642 (4)	147 (4)
C11—H11⋯O1^iv^	0.93	2.46	3.370 (5)	164
C14—H14*C*⋯O3^v^	0.96	2.58	3.425 (5)	147

Symmetry codes: (i) 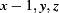; (ii) 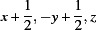; (iii) 
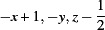
; (iv) 
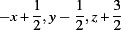
; (v) 
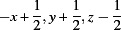
.

**Fig. 1.**
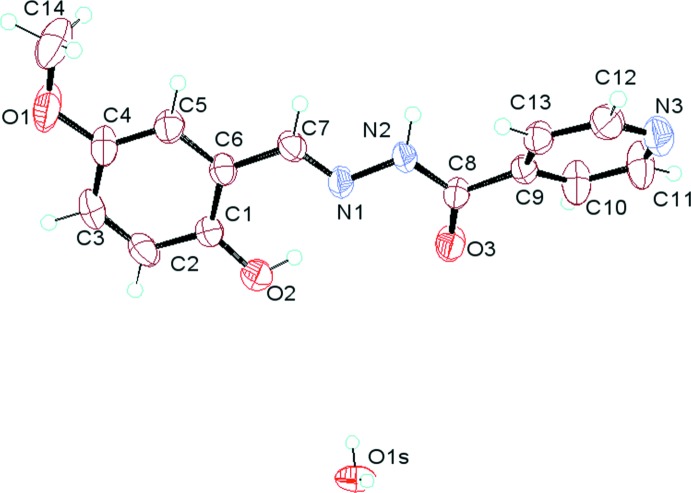
ORTEP view of the unique part of the compound, drawn with 50% probability displacement ellipsoids for the non-H atoms.

**Fig. 2.**
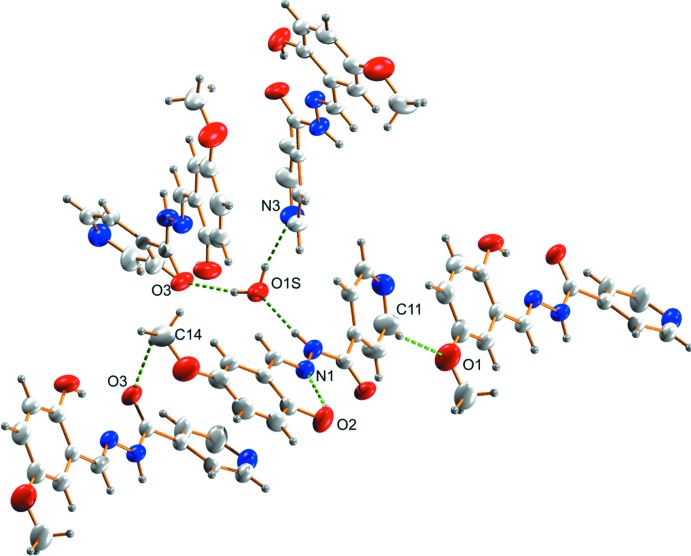
Graphical representation showing hydrogen bondings in the crystal structure of C14H13N3O3.H2O.

**Fig. 3.**
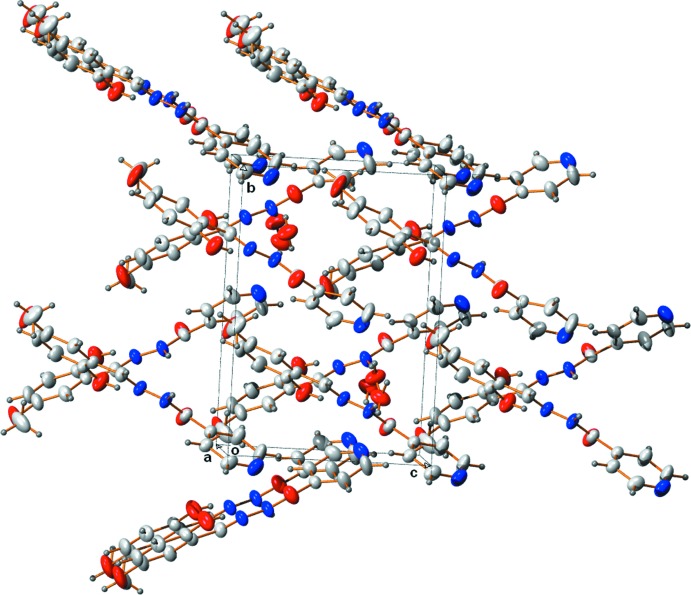
A view of the unit cell along a axis.

## supplementary crystallographic information

### Comment

Derivatives of carbohydrazide and their metal complexes exhibit prominent biological activities and versatile binding properties. They also promote the formation of a chelate binding center (Mangalam & Kurup, 2011). Versatile applications in molecular sensing of metals have received substantial interest during the last decade (Bakir & Brown, 2002).

The compound crystallizes in orthorhombic P*na*2_1_ space group. The molecule exists in the *E* configuration with respect to C7=N1 bond (Sithambaresan & Kurup, 2011) which is confirmed by the torsion angle of -177.6 (3)° of C6—C7—N1—N2 moiety (Fig. 1). The torsion angle of 7.2 (5)° corresponding to N1—N2—C8—O3 moiety supports the *cis* configuration of the O3 atom with respect to the hydrazine nitrogen atom N1 (Kargar *et al.*, 2010; Shafiq *et al.*, 2009). The C7=N1 [1.272 (4) Å] and C8=O3 [1.219 (3) Å] bond distances are very close to the formal C=N and C=O bond lengths [C=N; 1.28 Å and C═O; 1.21 Å] respectively confirming the azomethine bond formation and the existence of carbohydrazide in amido form in solid state.

Incorporation of water molecule in the crystal system of the title compound plays an important role in assembly of the molecules in the lattice through intermolecular hydrogen bonds and makes the crystal system entirely different from the reported one (Kargar *et al.*, 2010). Two classical hydrogen bonds are present in the molecular system (Fig. 2) between the both H atoms of the water molecule and O3 and N3 atoms of the neighbouring molecules with a D···A distances of 2.764 (4) and 2.874 (5) Å respectively. The phenolic oxygen O2 is involved in an intramolecular hydrogen bond with N1 to form a six membered ring. The hydrazinic nitrogen N2 is also involved in hydrogen bonding with oxygen of solvent water. Two non- classical C–H···O hydrogen bonds are also present in the molecule (Table 1). The packing diagram showing the molecular assembly of the title compound along *a* axis is shown in Fig. 3.

### Experimental

The title compound was prepared by adapting a reported procedure (Mangalam *et al.*, 2009). To a warm ethanolic solution of 2-hydroxy-5-methoxybenzaldehyde (0.152 g, 1 mmol), a methanolic solution of pyridine-4-carbohydrazide (0.137 g, 1 mmol) was added and the resulting solution was refluxed for 45 minutes after adding 3 drops of glacial acetic acid. On cooling the solution, yellow crystals separated out. Single crystals suitable for X-ray diffraction studies were obtained by slow evaporation of its solution in 1:1 mixture of ethanol and DMF.

### Refinement

All H atoms on C were placed in calculated positions, guided by difference maps, with C–H bond distances 0.93–0.96 Å. H atoms were assigned as *U*_iso_(H)=1.2Ueq(carrier) or 1.5Ueq (methyl C). N2–H2' and O2–H2A H atoms were located from difference maps and restrained using *DFIX* instructions. H atoms of the water molecule were also located from difference maps and restrained using *DFIX* and *DANG* instructions. Omitted owing to bad disagreement was the reflection (1 1 0). In the absence of significant anomalous scattering effects Friedel pairs have been merged.

### Crystal data

**Table:**

C_14_H_13_N_3_O_3_·H_2_O	*F*(000) = 608
*M**_r_* = 289.29	*D*_x_ = 1.335 Mg m^−^^3^
Orthorhombic, *P**n**a*2_1_	Mo *K*α radiation, λ = 0.71073 Å
Hall symbol: P 2c -2n	Cell parameters from 1943 reflections
*a* = 12.6455 (16) Å	θ = 2.8–27.5°
*b* = 12.7423 (16) Å	µ = 0.10 mm^−^^1^
*c* = 8.9306 (9) Å	*T* = 296 K
*V* = 1439.0 (3) Å^3^	Block, yellow
*Z* = 4	0.40 × 0.30 × 0.25 mm

### Data collection

**Table:**

Bruker Kappa APEXII CCD diffractometer	1668 independent reflections
Radiation source: fine-focus sealed tube	1134 reflections with *I* > 2σ(*I*)
Graphite monochromator	*R*_int_ = 0.056
Detector resolution: 8.33 pixels mm^-1^	θ_max_ = 27.0°, θ_min_ = 2.8°
ω and φ scan	*h* = −16→15
Absorption correction: multi-scan (*SADABS*; Bruker, 2004)	*k* = −14→16
*T*_min_ = 0.965, *T*_max_ = 0.976	*l* = −11→9
10294 measured reflections	

### Refinement

**Table:**

Refinement on *F*^2^	Secondary atom site location: difference Fourier map
Least-squares matrix: full	Hydrogen site location: inferred from neighbouring sites
*R*[*F*^2^ > 2σ(*F*^2^)] = 0.045	H atoms treated by a mixture of independent and constrained refinement
*wR*(*F*^2^) = 0.124	*w* = 1/[σ^2^(*F*_o_^2^) + (0.0701*P*)^2^] where *P* = (*F*_o_^2^ + 2*F*_c_^2^)/3
*S* = 1.00	(Δ/σ)_max_ < 0.001
1668 reflections	Δρ_max_ = 0.17 e Å^−^^3^
208 parameters	Δρ_min_ = −0.15 e Å^−^^3^
6 restraints	Extinction correction: *SHELXL97* (Sheldrick, 2008), Fc^*^=kFc[1+0.001xFc^2^λ^3^/sin(2θ)]^-1/4^
Primary atom site location: structure-invariant direct methods	Extinction coefficient: 0.012 (3)

### Special details

**Table:**

Geometry. All e.s.d.'s (except the e.s.d. in the dihedral angle between two l.s. planes) are estimated using the full covariance matrix. The cell e.s.d.'s are taken into account individually in the estimation of e.s.d.'s in distances, angles and torsion angles; correlations between e.s.d.'s in cell parameters are only used when they are defined by crystal symmetry. An approximate (isotropic) treatment of cell e.s.d.'s is used for estimating e.s.d.'s involving l.s. planes.
Refinement. Refinement of *F*^2^ against ALL reflections. The weighted *R*-factor *wR* and goodness of fit *S* are based on *F*^2^, conventional *R*-factors *R* are based on *F*, with *F* set to zero for negative *F*^2^. The threshold expression of *F*^2^ > σ(*F*^2^) is used only for calculating *R*-factors(gt) *etc*. and is not relevant to the choice of reflections for refinement. *R*-factors based on *F*^2^ are statistically about twice as large as those based on *F*, and *R*- factors based on ALL data will be even larger.

### Fractional atomic coordinates and isotropic or equivalent isotropic displacement parameters (Å^2^)

**Table:**

	*x*	*y*	*z*	*U*_iso_*/*U*_eq_	
O1	0.1808 (2)	0.4322 (3)	−0.0082 (3)	0.0751 (10)	
O2	0.4338 (2)	0.2150 (3)	0.3839 (3)	0.0617 (9)	
O3	0.37204 (18)	0.1124 (2)	0.7981 (3)	0.0518 (7)	
O1S	1.0079 (2)	0.2238 (2)	0.7337 (4)	0.0590 (8)	
N1	0.2710 (2)	0.2049 (2)	0.5685 (3)	0.0397 (7)	
N2	0.2196 (2)	0.1700 (3)	0.6956 (3)	0.0408 (7)	
N3	0.1091 (3)	−0.0404 (3)	1.1458 (4)	0.0575 (9)	
C1	0.3661 (3)	0.2681 (3)	0.2933 (4)	0.0411 (8)	
C2	0.4043 (3)	0.3044 (3)	0.1575 (4)	0.0507 (10)	
H2	0.4747	0.2928	0.1326	0.061*	
C3	0.3409 (3)	0.3566 (3)	0.0601 (4)	0.0533 (11)	
H3	0.3679	0.3792	−0.0312	0.064*	
C4	0.2361 (3)	0.3764 (3)	0.0963 (4)	0.0497 (10)	
C5	0.1962 (3)	0.3413 (3)	0.2291 (4)	0.0428 (9)	
H5	0.1257	0.3540	0.2528	0.051*	
C6	0.2605 (3)	0.2865 (3)	0.3301 (3)	0.0353 (8)	
C7	0.2147 (3)	0.2507 (3)	0.4707 (4)	0.0391 (8)	
H7	0.1432	0.2615	0.4890	0.047*	
C8	0.2760 (3)	0.1203 (3)	0.8009 (3)	0.0360 (8)	
C9	0.2130 (2)	0.0700 (3)	0.9218 (3)	0.0345 (8)	
C10	0.2574 (3)	0.0576 (3)	1.0612 (5)	0.0558 (11)	
H10	0.3236	0.0856	1.0823	0.067*	
C11	0.2026 (4)	0.0036 (4)	1.1683 (5)	0.0651 (13)	
H11	0.2329	−0.0025	1.2628	0.078*	
C12	0.0673 (3)	−0.0275 (3)	1.0122 (5)	0.0513 (10)	
H12	0.0012	−0.0569	0.9941	0.062*	
C13	0.1150 (3)	0.0265 (3)	0.8976 (4)	0.0404 (8)	
H13	0.0817	0.0336	0.8053	0.049*	
C14	0.0712 (4)	0.4431 (4)	0.0117 (5)	0.0759 (15)	
H14A	0.0391	0.3750	0.0175	0.114*	
H14B	0.0418	0.4810	−0.0714	0.114*	
H14C	0.0576	0.4809	0.1027	0.114*	
H2'	0.1551 (15)	0.191 (3)	0.720 (5)	0.054 (12)*	
H1A	0.973 (3)	0.279 (2)	0.757 (6)	0.093 (19)*	
H1B	0.962 (3)	0.175 (2)	0.722 (6)	0.081 (16)*	
H2A	0.400 (3)	0.198 (3)	0.462 (3)	0.066 (14)*	

### Atomic displacement parameters (Å^2^)

**Table:**

	*U*^11^	*U*^22^	*U*^33^	*U*^12^	*U*^13^	*U*^23^
O1	0.069 (2)	0.106 (3)	0.0501 (17)	−0.0069 (18)	−0.0104 (16)	0.0408 (18)
O2	0.0470 (15)	0.080 (2)	0.0580 (18)	0.0194 (14)	0.0147 (16)	0.0220 (16)
O3	0.0316 (13)	0.0695 (19)	0.0542 (15)	−0.0047 (12)	−0.0016 (12)	0.0243 (13)
O1S	0.0376 (14)	0.0544 (18)	0.085 (2)	0.0050 (14)	−0.0006 (15)	−0.0197 (16)
N1	0.0355 (15)	0.0484 (18)	0.0351 (15)	−0.0004 (14)	0.0036 (14)	0.0125 (14)
N2	0.0352 (16)	0.0528 (19)	0.0343 (16)	0.0034 (15)	0.0074 (14)	0.0151 (13)
N3	0.056 (2)	0.063 (2)	0.054 (2)	−0.0059 (18)	0.0109 (17)	0.0184 (17)
C1	0.0422 (19)	0.042 (2)	0.0394 (18)	0.0026 (16)	0.0076 (16)	0.0070 (16)
C2	0.046 (2)	0.060 (3)	0.046 (2)	−0.0011 (19)	0.0176 (18)	0.0028 (18)
C3	0.063 (3)	0.063 (3)	0.0331 (19)	−0.006 (2)	0.0128 (19)	0.0101 (18)
C4	0.055 (3)	0.058 (3)	0.036 (2)	−0.0101 (19)	−0.0029 (19)	0.0129 (19)
C5	0.0341 (18)	0.055 (2)	0.040 (2)	−0.0037 (16)	−0.0040 (16)	0.0079 (18)
C6	0.0357 (18)	0.037 (2)	0.0327 (18)	−0.0067 (14)	0.0004 (15)	0.0028 (15)
C7	0.0307 (18)	0.051 (2)	0.0357 (19)	−0.0024 (16)	0.0034 (15)	0.0033 (16)
C8	0.0342 (18)	0.0392 (19)	0.0346 (18)	−0.0041 (15)	−0.0024 (16)	0.0083 (15)
C9	0.0324 (17)	0.0366 (19)	0.0346 (18)	−0.0009 (14)	0.0022 (15)	0.0044 (15)
C10	0.054 (3)	0.070 (3)	0.044 (2)	−0.022 (2)	−0.011 (2)	0.017 (2)
C11	0.072 (3)	0.086 (4)	0.036 (2)	−0.021 (3)	−0.0063 (19)	0.023 (2)
C12	0.0378 (19)	0.053 (3)	0.063 (3)	−0.0039 (17)	0.0056 (19)	0.014 (2)
C13	0.0373 (18)	0.043 (2)	0.0413 (19)	−0.0026 (15)	−0.0012 (16)	0.0073 (16)
C14	0.066 (3)	0.086 (4)	0.076 (3)	−0.016 (3)	−0.030 (3)	0.036 (3)

### Geometric parameters (Å, º)

**Table:**

O1—C4	1.366 (4)	C3—H3	0.9300
O1—C14	1.405 (5)	C4—C5	1.364 (5)
O2—C1	1.358 (5)	C5—C6	1.402 (5)
O2—H2A	0.847 (10)	C5—H5	0.9300
O3—C8	1.218 (4)	C6—C7	1.456 (5)
O1S—H1A	0.856 (10)	C7—H7	0.9300
O1S—H1B	0.859 (10)	C8—C9	1.487 (4)
N1—C7	1.269 (4)	C9—C13	1.374 (5)
N1—N2	1.381 (4)	C9—C10	1.374 (5)
N2—C8	1.341 (4)	C10—C11	1.367 (6)
N2—H2'	0.885 (10)	C10—H10	0.9300
N3—C12	1.315 (5)	C11—H11	0.9300
N3—C11	1.325 (5)	C12—C13	1.373 (5)
C1—C2	1.384 (5)	C12—H12	0.9300
C1—C6	1.395 (5)	C13—H13	0.9300
C2—C3	1.357 (6)	C14—H14A	0.9600
C2—H2	0.9300	C14—H14B	0.9600
C3—C4	1.388 (6)	C14—H14C	0.9600
			
C4—O1—C14	118.0 (3)	N1—C7—H7	119.5
C1—O2—H2A	107 (3)	C6—C7—H7	119.5
H1A—O1S—H1B	106 (2)	O3—C8—N2	123.8 (3)
C7—N1—N2	116.7 (3)	O3—C8—C9	120.9 (3)
C8—N2—N1	118.5 (3)	N2—C8—C9	115.3 (3)
C8—N2—H2'	118 (3)	C13—C9—C10	117.7 (3)
N1—N2—H2'	122 (3)	C13—C9—C8	122.9 (3)
C12—N3—C11	116.3 (3)	C10—C9—C8	119.2 (3)
O2—C1—C2	117.9 (3)	C11—C10—C9	119.0 (4)
O2—C1—C6	123.1 (3)	C11—C10—H10	120.5
C2—C1—C6	118.9 (3)	C9—C10—H10	120.5
C3—C2—C1	121.3 (4)	N3—C11—C10	124.0 (4)
C3—C2—H2	119.3	N3—C11—H11	118.0
C1—C2—H2	119.3	C10—C11—H11	118.0
C2—C3—C4	120.2 (3)	N3—C12—C13	124.2 (4)
C2—C3—H3	119.9	N3—C12—H12	117.9
C4—C3—H3	119.9	C13—C12—H12	117.9
C5—C4—O1	125.1 (4)	C12—C13—C9	118.8 (3)
C5—C4—C3	119.8 (4)	C12—C13—H13	120.6
O1—C4—C3	115.1 (3)	C9—C13—H13	120.6
C4—C5—C6	120.5 (3)	O1—C14—H14A	109.5
C4—C5—H5	119.7	O1—C14—H14B	109.5
C6—C5—H5	119.7	H14A—C14—H14B	109.5
C1—C6—C5	119.2 (3)	O1—C14—H14C	109.5
C1—C6—C7	122.1 (3)	H14A—C14—H14C	109.5
C5—C6—C7	118.7 (3)	H14B—C14—H14C	109.5
N1—C7—C6	121.0 (3)		
			
C7—N1—N2—C8	179.3 (3)	C1—C6—C7—N1	3.1 (5)
O2—C1—C2—C3	179.0 (4)	C5—C6—C7—N1	−176.8 (3)
C6—C1—C2—C3	−0.4 (6)	N1—N2—C8—O3	7.1 (6)
C1—C2—C3—C4	1.1 (6)	N1—N2—C8—C9	−170.4 (3)
C14—O1—C4—C5	−8.9 (7)	O3—C8—C9—C13	−144.0 (4)
C14—O1—C4—C3	171.8 (4)	N2—C8—C9—C13	33.6 (5)
C2—C3—C4—C5	−1.3 (6)	O3—C8—C9—C10	30.2 (6)
C2—C3—C4—O1	178.1 (4)	N2—C8—C9—C10	−152.3 (4)
O1—C4—C5—C6	−178.6 (4)	C13—C9—C10—C11	−0.2 (6)
C3—C4—C5—C6	0.7 (6)	C8—C9—C10—C11	−174.6 (4)
O2—C1—C6—C5	−179.5 (4)	C12—N3—C11—C10	−1.9 (7)
C2—C1—C6—C5	−0.1 (5)	C9—C10—C11—N3	1.6 (7)
O2—C1—C6—C7	0.6 (5)	C11—N3—C12—C13	0.9 (6)
C2—C1—C6—C7	180.0 (3)	N3—C12—C13—C9	0.4 (6)
C4—C5—C6—C1	0.0 (5)	C10—C9—C13—C12	−0.7 (5)
C4—C5—C6—C7	179.9 (3)	C8—C9—C13—C12	173.5 (3)
N2—N1—C7—C6	−177.5 (3)		

### Hydrogen-bond geometry (Å, º)

**Table:**

*D*—H···*A*	*D*—H	H···*A*	*D*···*A*	*D*—H···*A*
N2—H2′···O1*S*^i^	0.89 (1)	1.91 (1)	2.784 (4)	168 (4)
O1*S*—H1*A*···O3^ii^	0.86 (1)	1.92 (2)	2.764 (4)	170 (4)
O1*S*—H1*B*···N3^iii^	0.86 (1)	2.05 (2)	2.874 (5)	160 (4)
O2—H2*A*···N1	0.85 (1)	1.89 (3)	2.642 (4)	147 (4)
C11—H11···O1^iv^	0.93	2.46	3.370 (5)	164
C14—H14*C*···O3^v^	0.96	2.58	3.425 (5)	147

Symmetry codes: (i) *x*−1, *y*, *z*; (ii) *x*+1/2, −*y*+1/2, *z*; (iii) −*x*+1, −*y*, *z*−1/2; (iv) −*x*+1/2, *y*−1/2, *z*+3/2; (v) −*x*+1/2, *y*+1/2, *z*−1/2.

## References

[bb1] Bakir, M. & Brown, O. (2002). *J. Mol. Struct.* **609**, 129–136.

[bb2] Brandenburg, K. (2010). *DIAMOND* Crystal Impact GbR, Bonn, Germany.

[bb3] Bruker (2004). *SADABS*, *APEX2*, *XPREP* and *SAINT* Bruker AXS Inc., Madison, Wisconsin, USA.

[bb4] Farrugia, L. J. (2012). *J. Appl. Cryst.* **45**, 849–854.

[bb5] Kargar, H., Kia, R., Akkurt, M. & Büyükgüngör, O. (2010). *Acta Cryst.* E**66**, o2982. 10.1107/S1600536810043382 PMC3009050 21589148

[bb6] Mangalam, N. A. & Kurup, M. R. P. (2011). *Spectrochim. Acta Part A*, **76**, 22–28.

[bb7] Mangalam, N. A., Sivakumar, S., Sheeja, S. R., Kurup, M. R. P. & Tiekink, E. R. T. (2009). *Inorg. Chim. Acta*, **362**, 4191–4197.

[bb8] Shafiq, Z., Yaqub, M., Tahir, M. N., Hussain, A. & Iqbal, M. S. (2009). *Acta Cryst.* E**65**, o2899. 10.1107/S1600536809044134 PMC2971391 21578481

[bb9] Sheldrick, G. M. (2008). *Acta Cryst.* A**64**, 112–122. 10.1107/S0108767307043930 18156677

[bb10] Sithambaresan, M. & Kurup, M. R. P. (2011). *Acta Cryst.* E**67**, o2972. 10.1107/S1600536811041857 PMC3247376 22219994

[bb11] Westrip, S. P. (2010). *J. Appl. Cryst.* **43**, 920–925.

